# Notch Signaling Activates Stem Cell Properties of Müller Glia through Transcriptional Regulation and Skp2-mediated Degradation of p27^Kip1^

**DOI:** 10.1371/journal.pone.0152025

**Published:** 2016-03-24

**Authors:** Carolina Beltrame Del Debbio, Qulsum Mir, Sowmya Parameswaran, Saumi Mathews, Xiaohuan Xia, Li Zheng, Andrew J. Neville, Iqbal Ahmad

**Affiliations:** Department of Ophthalmology and Visual Sciences, University of Nebraska Medical Center, Omaha, Nebraska, United States of America; National Eye Institute, UNITED STATES

## Abstract

Müller glia (MG), the sole glial cells generated by retinal progenitors, have emerged as a viable cellular target for therapeutic regeneration in degenerative blinding diseases, as they possess dormant stem cell properties. However, the mammalian MG does not display the neurogenic potential of their lower vertebrate counterparts, precluding their practical clinical use. The answer to this barrier may be found in two interlinked processes underlying the neurogenic potential, i.e., the activation of the dormant stem cell properties of MG and their differentiation along the neuronal lineage. Here, we have focused on the former and examined Notch signaling-mediated activation of MG. We demonstrate that one of the targets of Notch signaling is the cyclin-dependent kinase inhibitor (CKI), p27^Kip1^, which is highly expressed in quiescent MG. Notch signaling facilitates the activation of MG by inhibiting p27^Kip1^ expression. This is likely achieved through the Notch- p27^Kip1^ and Notch-Skp2-p27^Kip1^ axes, the former inhibiting the expression of *p27*^*Kip1*^ transcripts and the latter levels of p27^Kip1^ proteins by Skp2-mediated proteasomal degradation. Thus, Notch signaling may facilitate re-entry of MG into the cell cycle by inhibiting p27^Kip1^ expression both transcriptionally and post-translationally.

## Introduction

The retina, an integral part of the central nervous system (CNS), does not display active neurogenesis under normal conditions in adult mammals. However, when injured it displays neurogenic potential, which can be traced to the major glial cell type of the retina, MG [1–3]. Emerging evidence supporting this property of MG has placed them in a similar category as the radial glia in the CNS, where they sub serve the function of neural stem cells [4]. Recent evidence supports this notion. For example, MG enriched from the mammalian retina displayed central features of neural stem cells, i.e., the ability to self-renew and differentiate along both neuronal and glial lineages [5]. Examination of the neurotoxin-damaged chick and mammalian retina revealed a rare subset of activated MG that had incorporated BrdU and expressed markers corresponding to retinal neurons [6–10]. Furthermore, when activated MG, prospectively enriched from the neurotoxin-damaged retina, were transplanted they integrated in the host retina and expressed markers corresponding to rod photoreceptors and retinal ganglion cells (RGCs) in the outer nuclear layer (ONL) and RGC layer, respectively [5]. However, despite the evidence of neuronal conversion of the mammalian MG, the efficiency of their neurogenic potential significantly lags behind their lower vertebrate counterparts, such as teleosts, in which retina is regenerated throughout the life. For example, using a variety of injury models and transgenic zebrafish for lineage analyses, various labs have shown an efficient conversion of MG into retinal neurons, particularly photoreceptors [11–13].

These studies demonstrated that the neurogenic property of MG, albeit reduced in mammals, is evolutionarily conserved and may be tapped into for therapeutic regeneration of the mammalian retina [1–3]. Recently, this notion was tested in a rat model of retinitis pigmentosa, where MG activated by Notch and Wnt signaling and tagged by BrdU/genetic markers were observed to have migrated to the ONL and a rare subset of these migrated MG expressed markers corresponding to rod photoreceptors [14]. To understand the reduced neurogenic potential of the mammalian MG, we have begun examining the two interlinked processes underlying the potential, i.e., the activation and neural conversion of MG. Here, we have focused on the former, specifically, how Notch signaling interacts with the intrinsic machinery of MG to regulate their activation. Notch signaling plays an integral role in differentiation of MG during development [15–19], and in their activation in response to injury both in mammals [5] and lower vertebrates [20,21]. Activation of the Notch receptor leads to gamma secretase-mediated release of the Notch intracellular domain (NICD) [22]. NICD translocates to the nucleus where it recruits a co-activator complex, consisting of CSL (CBF1, suppressor of hairless, and Lag1) and Mastermind-like (MAML1), that binds to genes containing the CSL binding sites, activating their transcription. The *Hes* family of genes, a primary target of Notch signaling, encodes transcriptional repressors that attenuate the expression of their target genes by binding to C-box/N-box elements in their promoters. Since Notch signaling induces proliferation in mitotically quiescent MG [3,5], cyclin-dependent kinase inhibitors (CKIs), which regulate cell cycle progression by inhibiting the phosphorylation of retinoblastoma proteins by G1-phase cyclins [23], emerge as potential Notch targets in MG. This premise is supported by the observations that CKIs are targeted by Notch signaling and are known to coordinate cell cycle exit with self-renewal properties of stem cells [24, 25]. Among the CKIs, p27^Kip1^ is likely to be the key regulator of the G1/S transition and S phase progression of MG because, unlike other CKIs such as p57^Kip2^ and p21^Cip1^, it is expressed at high levels in adult MG [26,27]. Additionally, its expression is inversely co-related with that of its proteasomal regulator, Skp2, which is positively regulated by Notch signaling [24]. Based on the previous observations that p27^Kip1^ keeps MG from entering the cell cycle [28], we hypothesized that Notch signaling-dependent inhibition of p27^Kip1^ constitutes a key event in the activation of MG. The test of the hypothesis revealed that Notch signaling regulates the activation of MG by influencing two different Notch-dependent intracellular axes, namely, Notch-p27^Kip1^ and Notch-Skp2-p27^Kip1^. Thus, Notch signaling facilitates re-entry of MG into the cell cycle by inhibiting p27^Kip1^ expression, both transcriptionally and post-translationally.

## Materials and Methods

### Animals

All experiments were conducted in accordance with the Association for Research in Vision and Ophthalmology (ARVO) Statement for the Use of Animals in Ophthalmic and Vision Research, and were approved by the Institutional Animal Care and Use Committee (IACUC) at University of Nebraska Medical Center (protocols: # 95-005-09 and # 97-100-08). Various ages of Sprague-Dawley (SD) rats, beginning from embryonic day 18 (E18) to postnatal day 21 (PN21), were purchased from Charles River Laboratories (Wilmington, MA). Animals were housed in the Department of Comparative Medicine at the University of Nebraska Medical Center. Rats were euthanized by CO_2_ exposure followed by decapitation using sterile surgical scissors to ensure death.

### Enrichment of MG and neurosphere assays

MG were enriched from the retinas of 10–12 PN10 SD rats/batch of analysis using the hypoxia-based method and subsequently analyzed for MG enrichment [5]. Neurosphere assays were carried out as previously described [5,14]. Briefly, the enriched MG were cultured in the presence of retinal culture medium (RCM = DMEM-F12, 1X N2 supplement, 2mM L-glutamine, 100 U/ml penicillin, 100 μg/ml streptomycin), FGF2 (20 ng/ml), Heparin (2 μg/ml), and either Jag1 peptide (20μM, to activate Notch signaling) and/or DAPT (20μM, to inactivate Notch signaling) for 5 days. For the loss-of-function (LOF) assays, the enriched MG were transduced with *p27*^*Kip1*^ or *Skp2* siRNA lentivirus or control lentivirus before the neurosphere assays.

### Viral vectors and lentivirus production

The *p27*^*Kip1*^ shRNA lentivirus construct was obtained from Thermo Scientific (Waltham, MA). The *Skp2* siRNA lentivirus construct was obtained from Applied Biological Materials Inc. (ABM, Richmond, BC, Canada). The control GFP lentivirus construct was obtained from Addgene (Cambridge, MA). In the *p27*^*Kip1*^ shRNA and *Skp2* siRNA lentivirus constructs, the expression of sh/siRNA sequences were driven by U6 promoters. In GFP lentivirus constructs, the CMV promoter drove expression of the GFP sequence. Lentivirus preparation and transduction were as previously described [27]. Briefly, the recombinant lentiviral particles were generated using the ABM lentivirus packaging system, followed by transient transfection of 293T cells, and subsequently concentrated using BioVision PEG lentivirus precipitation kit (Milpitas, CA). Virus titer was determined using the ABM lentivirus titration kit. Enriched MG and retinal explants were transduced with lentiviruses with the multiplicity of infection (MOI) of 2–4, overnight. Viruses were removed the following morning. The specificity of LOF assays by virus-mediated sh/siRNA expression was determined in C6 glioma cells by Western and Q-PCR analyses. The efficiency of transduction in MG was ascertained by simultaneously co-transducing sh/siRNA and GFP lentivirus, where the GFP^+^ cells should reflect the sh/siRNA transduction efficiency.

### Explant assays

The explant assays were carried out as previously described [14]. Briefly, retinas removed from PN21 SD rats were placed on a semi-permeable membrane (0.4 mm pore size; Millipore, Temecula, California, USA), with the ganglion cell layer (GCL) oriented upward, and were maintained in RCM with 5% fetal bovine serum (FBS; Hyclone, Canada). Explants were transduced with *p27*^*Kip1*^ shRNA or *Skp2* siRNA or control lentivirus (overnight, with removal of viral media the following morning) and cultured in the presence of Jag1 (20μM) or Jag1+MG132 (2μM) for 5 days. Before explants were collected for analyses on day 5, they were exposed to 5-Bromo-2-deoxyuridine (BrdU, Sigma Aldrich, St Louis, MO, USA, 10μM) for 8 hours. To determine the efficiency of the LOF approach, explants were simultaneously co-transduced with a GFP lentivirus and either *p27*^*Kip1*^ shRNA or *Skp2* siRNA lentivirus.

### Cell cycle analysis by flow cytometry

Cell cycle analysis of activated MG was carried out as previously described [29]. Briefly, cells were fixed in 70% ethanol at 4°C for 30 minutes, followed by incubations at 4°C for 30 minutes in Telford reagent containing the DNA-intercalating dye, propidium iodide. Data were acquired by flow cytometry and analyzed by ModFitLT V3.3.11 (Windows).

### Quantitative polymerase chain reaction (qPCR)

Total RNA was isolated using Mini RNeasy Kit (Qiagen, Hilden, Germany), and cDNA was synthesized as previously described [30]. Specific transcripts were amplified with gene-specific forward and reverse primers (Table 1) by using Quantifast SYBR Green PCR kit (Qiagen, Hilden, Germany) and a RotorGene 6000 (Corbett Robotics, San Francisco, CA, USA). Results obtained from qPCR were generated by measuring each sample in triplicate; no-template blanks were used as negative controls. Amplification curves and gene expressions were normalized to the housekeeping gene, *glyceraldehyde phosphate dehydrogenase* (*GAPDH*).

**Table 1:** Primers used for qPCR and ChIP analyses.

Gene	Primer sequence (5’—3’)	Product size (bp)	Annealing temp. (°C)
*GS*	FW: TCACAGGGACAAATGCCGAG; RV: GTTGATGTTGGAGGTTTCGTGG	362	58
*GAPDH*	FW: ACAGTCCATGCCATCACTGCC; RV: GCCTGCTTCACCACCTTCTTG	266	60
*Hes1*	FW: CCTCTCCTTGGTCCTGGAATAG; RV: AGGCTGTCTTTGGTTTGTCCG	281	54
*Nestin*	FW: TGGAGCAGGAGAAGCAAGGTCTAC; RV: TCAAGGGTATTAGGCAAGGGGG	295	56
*p27*^*Kip1*^	FW: TGTAGTGTCCTTTCGGTGAGAACTG; RV: GAATCTTCGGAACTCCCAAATGAG	126	50
*Skp2*	FW: ATGGACTGCTCTCAAACCTCGG; RV: GGAAACACCTGGAAAGTTCTCTCG	131	55
*Sox9*	FW: TGGAAACTTCAGTGGGAGCG; RV: AAACAGAGAACGAAACCAGGGC	101	52.5
*p27*^*Kip1*^ promoter (C-site)	FW: AGAGCAGGTTTGTTGGCAGT; RV: AGAGGAGGAGCTCCATTGGT	194	56
*CSL* binding site 1	FW: TCAGGGATCCTTCAGTCTCG; RV: TCCAAATACCCACAACTCCCC	193	56
*CSL* binding site 2	FW: GGTATTTGGAGGGTTCGTCC; RV: TAGCAACGTTCCATCACCAA	172	54

FW: Forward primer. RV: Reverse primer. *GS*: *Glutamine synthetase*.

### Chromatin immunoprecipitation (ChIP) analysis

ChIP analysis was carried out as previously described [31]. Antibody-specific information for ChIP (as well as Western and immunofluorescence analyses) can be found in Table 2. Briefly, cells were first cross-linked and serially quenched with 1% formaldehyde and glycine, respectively, followed by immunoprecipitation using anti-Hes1/anti-CSL antibodies and Chromatin Immunoprecipitation kit (Magna CHIP HiSens, Millipore, Germany) as per manufacturer’s instruction. For controls, immunoprecipitation was carried out with specific immunoglobulin G (IgG) antibodies. After proteinase and RNAse A digestion, the precipitated DNA was purified using a Qiaquick PCR purification kit (Qiagen Inc). Regular PCR was carried out using promoter-specific primers (Table 1), followed by the resolution of the PCR products by gel electrophoresis.

**Table 2:** Antibodies used in immunofluorescence and Western analyses.

Primary antibody	Application(s)	Blocking solution	Antibody dilution^a^
mAb mouse anti-**GS** (Millipore; cat# MAB302)	ICC/IHC	5% NGS and 0.02% Triton-X in 1X PBS	1:100
pAb rabbit anti-**GS** (Sigma; cat# G2781)	WB	5% non-fat dry milk in TBS-T (0.05%, Tween-20) + 1% BSA	1:5000
mAb mouse anti-**p27**^**Kip1**^ (Abcam; cat# ab3928)	ICC/IHC/WB	ICC & IHC: 5% NGS and 0.04% Triton-X in 1X PBS; WB: 5% non-fat dry milk in TBS-T (0.05%, Tween-20) + 1% BSA	1:2000
mAb rabbit anti-**Hes1** (Cell Signaling; cat# 11988S)	ICC	5% NGS and 0.04% Triton-X in 1X PBS	1:200
pAb rabbit anti-**Skp2** (Invitrogen; cat# 51–1900)	ICC/IHC/WB	ICC & IHC: 5% NGS and 0.04% Triton-X in 1X PBS; WB: 5% non-fat dry milk in TBS-T (0.05%, Tween-20) + 1% BSA	1:200
pAb rabbit anti-**Notch1** (Abcam; cat# ab27526)	ICC	5% NGS and 0.04% Triton-X in 1X PBS	1:100
mAb mouse anti-**Nestin** (Developmental Studies Hybridoma Bank; cat# Rat 401)	ICC	5% NGS and 0.02% Triton-X in 1X PBS	1:4
pAb rabbit anti-**Sox9** (Millipore; cat# Ab5535)	ICC/IHC	5% NGS and 0.04% Triton-X in 1X PBS	1:200
mAb mouse anti-**GAPDH** (Ambion; cat# 4300)	WB	5% non-fat dry milk in TBS-T (0.05%, Tween-20) + 1% BSA	1:6000
pAb rabbit anti-**NICD** (Abcam; cat# ab8925)	ICC/WB	ICC: 5% NGS and 0.04% Triton-X in 1X PBS; WB: 5% non-fat dry milk in TBS-T (0.05%, Tween-20) + 1% BSA	ICC: 1:100; WB: 1:500
pAb rabbit anti-**GLAST** (Abcam; cat# ab416)	ICC/IHC	5% NGS and 0.02% Triton-X in 1X PBS	1:100
Rat anti-**BrdU** (Accurate Chemical Co.; cat# OBT0030)	IHC/ICC	5% NGS and 0.04% Triton-X in 1X PBS	1:100
pAb Normal Rabbit **IgG** (Cell Signaling; cat# 2729S)	ChIP	5% non-fat dry milk in TBS-T (0.05%, Tween-20) + 1% BSA	1:200
pAb rabbit anti-**CSL** (Millipore; cat# Ab5790)	ChIP	5% non-fat dry milk in TBS-T (0.05%, Tween-20) + 1% BSA	1:200

ICC: Immunocytochemistry. IHC: Immunohistochemistry. WB: Western blot. NGS: Normal goat serum. mAb: Monoclonal antibody. pAb: Polyclonal antibody. GS: Glutamine Synthetase. GAPDH: glyceraldehyde phosphate dehydrogenase. NICD: Notch intracellular domain. GLAST: Glutamate-aspartate transporter. BrdU: 5-Bromo-2´-Deoxyuridine. PBS: Phosphate buffered saline. ChIP: Chromatin immunoprecipitation. TBS-T: Tris-buffered-saline + Tween-20. BSA: Bovine serum albumin.

^a^Unless specifically stated, an antibody used for multiple applications had the same dilution for each application.

### Immunofluorescence analysis

Immunofluorescence analysis was performed as previously described [5]. Briefly, 4% paraformaldehyde-fixed cells or explant cryosections (12μm thickness) were blocked in blocking solution, followed by an overnight incubation with specific antibodies (Table 2). Cells were examined for epifluorescence after incubation with anti–species-specific IgG conjugated to Cy3 or FITC (Life Technologies). Samples were mounted using VectaShield (Vector Laboratories, Burlingame, CA) and images were taken using a Zeiss AX10 fluorescence microscope and AxioVision Rel. 4.8 software. For quantification of the percentage of specific cell types in each experiment, the numbers of cell-specific antigen-positive cells were counted in 15 randomly selected fields (3 cover slips, 5 fields each), in triplicate. Adobe Photoshop CS4 was used for image presentation. Manipulation of the images was restricted to threshold and brightness adjustments to the whole image. Controls for the experiments consisted of the omission of primary antibodies. No staining was observed in these cases.

### Western analysis

Protein samples (10–50μg), extracted from cultured cells (pooled neurospheres from 4–5 T75 flasks/group; pooled neurospheres from 2 T75 flasks/group in triplicates) using T-PER Tissue Protein Extraction Reagent (Thermo Scientific, Waltham, MA), were denatured and separated by sodium dodecyl sulfate-polyacrylamide gel (12%) electrophoresis, and transferred onto a 0.45 micron PVDF-Plus Transfer Membrane (GE Water & Process Technologies). Membranes were blocked for 2 hours at room temperature in TBS-Tween (25mM Tris-HCl, pH 8.2, 144mM NaCl, 0.1% Tween-20) containing 5% skim milk and 1% BSA, followed by overnight incubation at 4°C with primary antibodies (Table 2). Membranes were washed and incubated with HRP-conjugated secondary antibodies for 1 hour at room temperature, and visualized with an enhanced chemiluminescence reagent (ECL Plus Western Blotting Detection System, Amersham) using Syngene gel documentation system (Fredrick, MD, USA). Immunoreactive bands corresponding to p27^Kip1^, Skp2, NICD, and GAPDH (loading control) were scanned using GeneTooLs image analysis software (Syngene) with minimal manual input and automated detection and compensation for the background and band distortion. Adobe Photoshop CS4 was used for image presentation. Manipulation of the images was restricted to equal brightness adjustments to all rows.

### Hoechst dye efflux assay (HDEA)

Neurospheres were dissociated into single cells and Hoechst dye efflux assays (HDEA) were carried out as previously described [5,31]. Neurosphere dissociates were suspended in Hoechst Iscove’s modified Dulbecco’s medium (IMDM) supplemented with 2% FBS and 1mM HEPES at 4°C overnight. Cells were stained with Hoechst 33342 (3.0 μg/ml) for 30 min at 37°C. Verapamil (100 μM), an inhibitor of the efflux pump called the ABCG2 transporter, and propidium iodide were used as negative and dead cell controls, respectively. The SP and non-SP regions were defined on Hoechst-low and Hoechst–bright, based on the Hoechst blue and red axes, respectively.

### Statistical analysis

Cell type-specific antigen-positive cells were counted in 10–15 randomly selected fields in three to five different coverslips (cell culture) or sections (explants). Each experiment was repeated at least three times. Values were expressed as mean ± SEM. Data were analyzed using the Student’s t-test or ANOVA to determine the significance of the differences between treatment and control groups. QPCR results were based on the individual gene expression in comparison to the respective control of the experimental group.

## Results

### Evidence of Notch-dependent regulatory axes during late retinal histogenesis and MG activation

**Fig 1:**
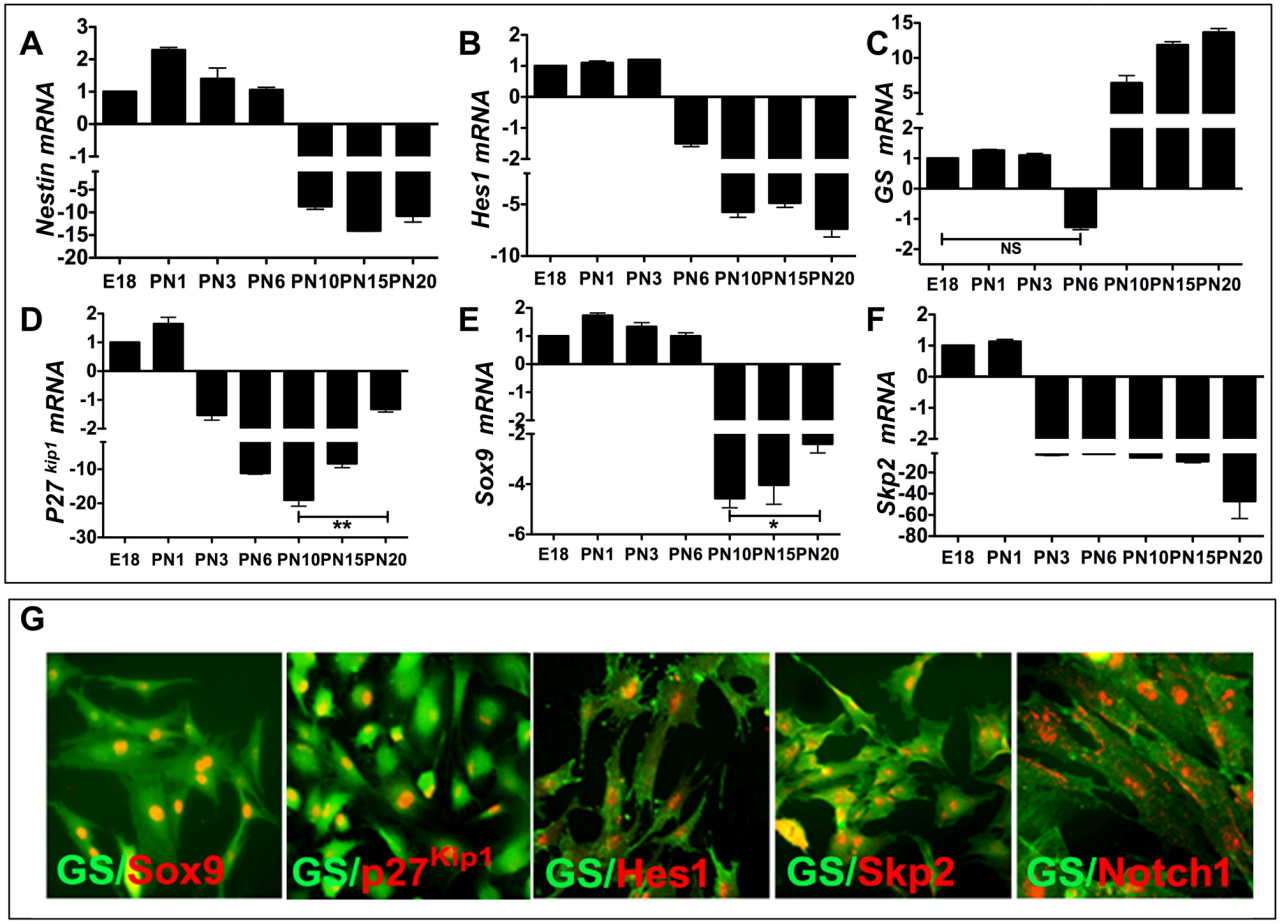
Expression of the Notch-dependent regulatory axes’ components during late retinal histogenesis and in enriched MG. **(A-C)** Levels of transcripts (fold change with respect to those at E18) corresponding to *Nestin/Hes1* and *GS* temporally decreased and increased, respectively, during the stages of late retinal histogenesis (E18-PN10) and beyond (PN15-PN20). **(D-F)** Levels of transcripts corresponding to *p27*^*Kip1*^, *Sox9*, and *Skp2* progressively decreased during late histogenesis, except that those of *p27*^*Kip1*^ and *Sox9* significantly increased at PN20, compared to levels at PN10. **(G)** Immunocytochemical analysis of enriched MG revealed that GS^+^ cells co-expressed immunoreactivities corresponding to p27^Kip1^, Skp2, Hes1, and NICD (activated Notch1). *p<0.05; **p<0.005; NS = non significant. Data are mean ± SEM.

To determine whether or not the proposed Notch-dependent regulatory axes exist during late retinal histogenesis, when MG are generated, we examined the temporal patterns of expression of the axes’ components against those encoding the regulators of progenitors and MG. During late histogenesis, retinal progenitors are progressively depleted as they differentiate into late born retinal cells. Accordingly, levels of transcripts corresponding to *Nestin*, a neural progenitor marker, and *Hes1*, the Notch-dependent regulator of retinal progenitors, decreased with time, compared to those at the beginning of late histogenesis, i.e., the embryonic day 18 (E18) stage (Fig 1A and 1B). In contrast, a reciprocal temporal increase in levels of MG-specific transcripts, *glutamine synthetase* (*GS*), was observed, demonstrating the expected inverse temporal relationship between the loss and gain of progenitor and MG phenotypes, respectively (Fig 1C). Next, we examined the temporal expression patterns of *p27*^*Kip1*^ and *Sox9*, both associated with retinal progenitors during retinal histogenesis and matured MG [28, 32–36]. Levels of *p27*^Kip1^ and *Sox9* transcripts decreased with time, however, the trend in temporal decrease plateaued around PN10 and increased there after such that significant transcript levels were detected at PN20, compared to those at PN10 (Fig 1D and 1E). Levels of transcripts corresponding to *Skp2*, a direct target of Notch signaling [24], temporally decreased in parallel with the attenuation in the levels of *Hes1* (Fig 1F). These observations suggested that the components of Notch-dependent regulatory axes, i.e., *Notch-p27*^Kip1^ and *Notch-Skp2-p27*^Kip1^, were expressed during late retinal histogenesis, which may be recruited for MG activation. We tested this hypothesis in enriched MG culture, a robust model for testing the activation and progenitor properties of MG when coupled with neurosphere assays [5, 14]. We first examined the expression of the components of the proposed axes in enriched MG; immunocytochemical analysis revealed glutamine synthetase (GS)^+^ MG co-expressing immunoreactivities corresponding to Sox9, p27^Kip1^, Hes1, Skp2, and NICD (activated Notch1) (Fig 1G).

**Fig 2:**
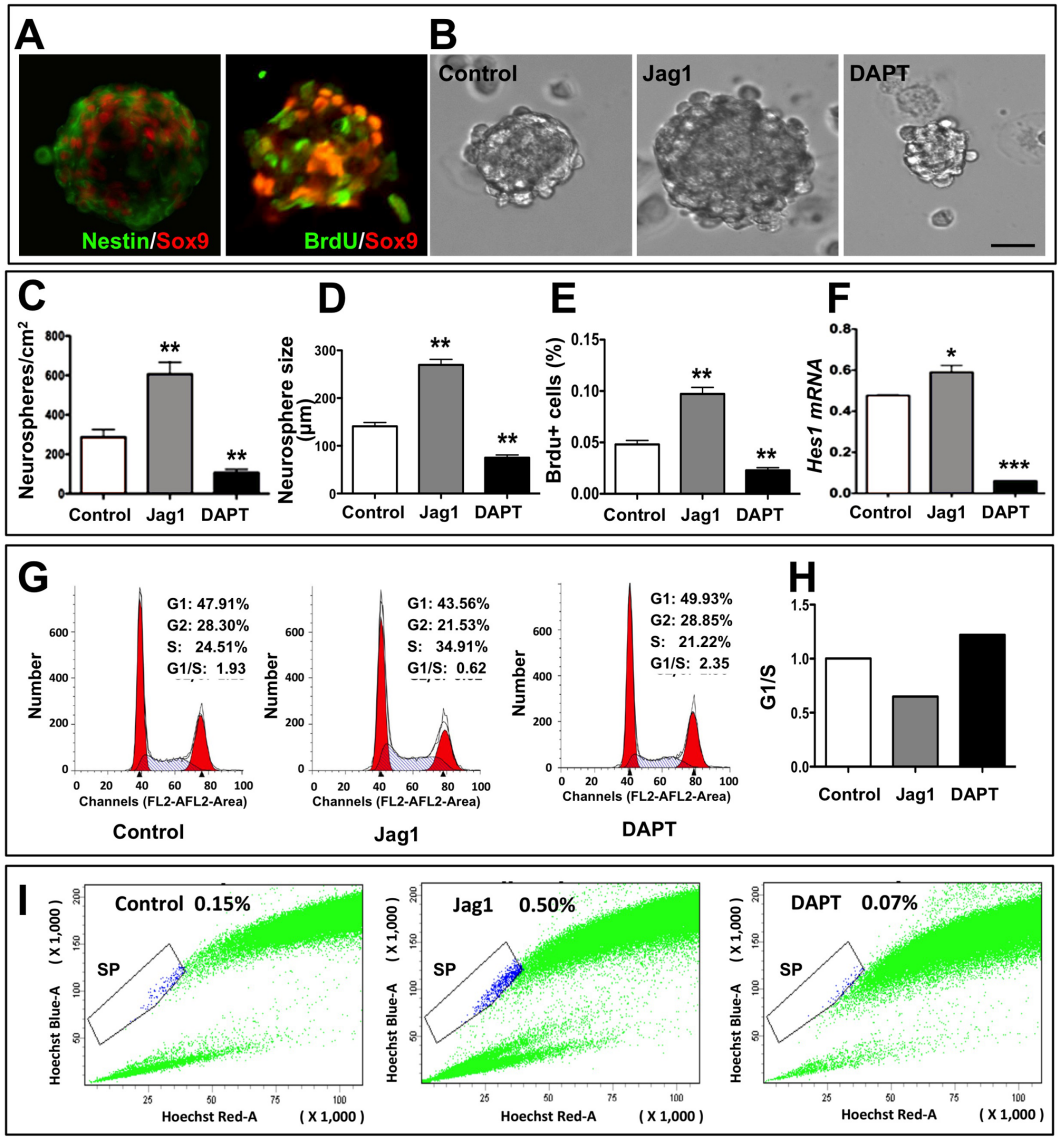
Effects of perturbation of Notch signaling in MG-derived neurospheres assays. **(A)** Immunofluorescence analysis of MG-derived neurospheres revealed Sox9^+^ cells, most of which co-expressed Nestin immunoreactivities or incorporated BrdU, suggesting their progenitor character. **(B)** Activation of Notch signaling by Jag1 in MG generated larger neurospheres compared to controls. In contrast, smaller neurospheres were generated with DAPT treatment. **(C-F)** A significant increase and decrease in the number (c) and size (d) of neurospheres and BrdU+ cells therein (e) was observed in Jag-1 and DAPT-treated groups, respectively, compared to controls. *Hes1* transcripts levels (f) increased and decreased in the presence of Jag1 and DAPT, respectively, compared to controls, confirming perturbation of Notch signaling. **(G, H)** The ratio of the proportion of cells in G1 and S phases increased in DAPT treated MG neurospheres, revealing the influence of Notch signaling on cell cycle during neurospheres formation (g). The profile of the G1/S ratio suggested the arrest to be in the G1 phase (h). **(I)** Hoechst dye efflux assay revealed more than triple the amount of SP cells in Jag1-treated MG neurospheres compared to control. Contrarily, DAPT treatment halved the amount of SP cells, compared to the control.*p<0.05 and **p<0.005. Scale bar = 100 μm. Data are mean ± SEM. Experiments were carried out three times in triplicates with 10–12 PN10 rats for MG enrichment/batch.

Next, we examined the perturbation of Notch signaling in FGF2-mediated neurosphere assays [5]. To activate Notch signaling, we cultured MG in the presence of Jag1, a 17-amino-acid peptide, corresponding to a region in the CSL domain of Notch ligand, Jagged1 (Jag1) [14]. To attenuate Notch signaling, MG were cultured in the presence of DAPT, a small molecule that compromises the generation of NICD by inhibiting gamma-secretase [14]. By the end of five days in control culture conditions, a subset of MG generated neurospheres (Fig 2). Cells in neurospheres expressed Nestin and Sox9, and incorporated BrdU, thus displaying their progenitor nature (Fig 2A). However, when MG were cultured in the presence of Jag1, they generated neurospheres which were significantly larger and more numerous, compared to controls (Fig 2B, 2C and 2D). In contrast, when DAPT was added to the culture medium, MG gave rise to significantly smaller and fewer neurospheres, compared to those in either Jag1 group or controls (Fig 2B, 2C and 2D). Quantification of neurosphere cells after 8 hour exposure to BrdU in the culture medium revealed a significant increase and decrease in the number of BrdU^+^ cells in the Jag1 and DAPT groups, respectively, versus controls (Fig 2E), corroborating effects of Jag1/DAPT on MG proliferation as ascertained by changes in the frequency and size of neurospheres. That the effects on MG proliferation were due to the perturbation in Notch signaling was demonstrated by a relative increase and decrease in the levels of *Hes1* transcripts in the presence of Jag1 and DAPT, respectively, compared to controls (Fig 2F). The effects of Notch signaling on MG proliferation is likely due to its influence on cell cycle regulation [24, 25]. To test this premise, we examined the effects of perturbation in Notch signaling on cell cycle by flow cytometry. We observed a relative increase in the proportions of cells in the S phase in Jag1 group versus controls, which decreased remarkably when cells were exposed to DAPT (Fig 2G). The increase in the ratio of the proportion of cells in G1/G2 and S phase between Jag1 and DAPT groups revealed that DAPT led to the arrest of MG at both G1 and G2 phases, suggesting the influence of Notch signaling on cell cycle regulation during the activation of MG (Fig 2H). Next, we were interested in knowing whether or not Notch signaling influenced the dormant stem cell properties of the activated MG. Another attribute, besides the capacity to generate clonal neurospheres of activated MG, is their ability to emerge as side population (SP) cells when subjected to the Hoechst dye efflux assay (HDEA) [5]. SP cell phenotype is a universal characteristic of stem cells/progenitors [37–39]. We observed a relative increase in the proportion of MG-SP cells in the presence of Jag1 versus controls, which decreased in the presence of DAPT (Fig 2G). Together, these results suggested that Notch signaling influenced the proliferation, cell cycle regulation, and stem cell properties of the activated MG.

### Influence of Notch signaling on *p27*^*Kip1*^ and *Skp2* expressions during MG activation

**Fig 3:**
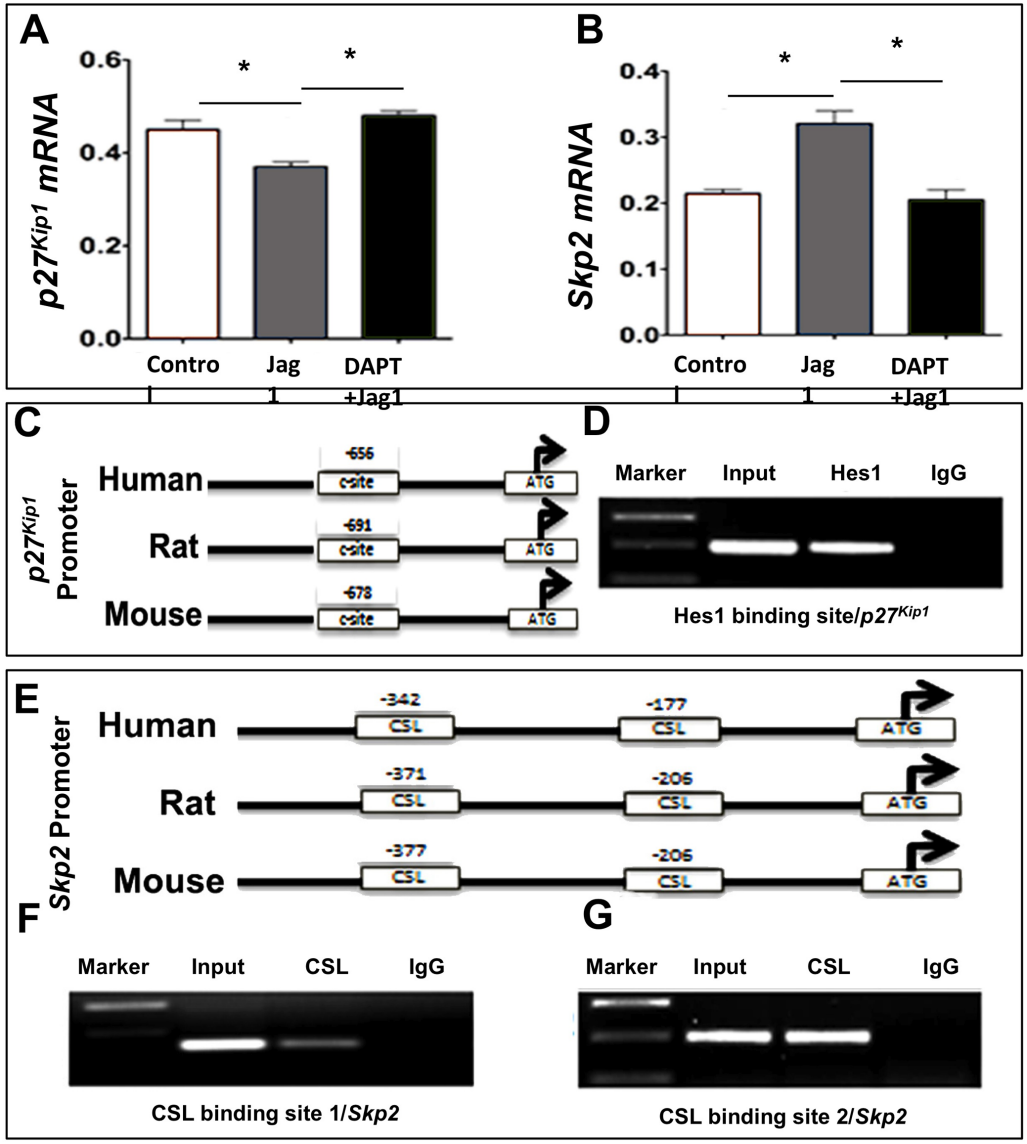
Influence of Notch signaling on p27^kip1^ and Skp2 expressions. **(A, B)** Activation of Notch signaling (Jag1) significantly decreased and increased levels of *p27*^*kip1*^ (a) and *Skp2* (b) transcripts, respectively, compared to controls. Their levels were recovered to that in controls in the presence of DAPT. **(C)** Schematic representation of *p27*^*kip1*^ proximal promoter containing an evolutionarily conserved C-box (C-site) element. **(D)** ChIP analysis carried out on enriched MG using Hes1 antibody revealed a sequence corresponding to *Hes1* promoter containing the C-site, as demonstrated by the size and sequence specific PCR products. **(E)** Schematic representation of *Skp2* proximal promoter containing evolutionarily conserved CSL binding sites. **(F)** ChIP analysis carried out on enriched MG using CSL antibody revealed sequences corresponding to CSL promoter containing the CSL sites, as demonstrated by the size and sequence specific PCR products. The specificity of the ChIP assays was demonstrated by the absence of PCR products in the IgG controls. *p<0.05. Data are mean ± SEM.

Next, we examined whether or not Notch signaling influenced the expression of *p27*^*Kip1*^ and *Skp2* during the activation of MG. Since the premise required Notch signaling to influence their expression at transcriptional and post-translational levels, we examined both their transcript and protein expressions. We first determined the effects of Notch signaling on levels of *p27*^*Kip1*^ and *Skp2* transcripts. We observed that the levels of transcripts corresponding to *p27*^*Kip1*^ and *Skp2* decreased and increased significantly in MG-derived neurospheres exposed to Jag1, respectively, compared to controls (Fig 3A and 3B). Exposure of neurospheres to DAPT abrogated the effects of Jag1 on the expression of *p27*^*Kip1*^ and *Skp2*; DAPT significantly increased and decreased *p27*^*Kip1*^ and *Skp2* transcript levels, respectively, compared to those in Jag1-treated groups, thus restoring to those in controls. The lack of significant difference in *p27*^*Kip1*^ and *Skp2* transcript levels, when compared between controls and DAPT groups, is likely due to the stability of NICD in glial cells (see below) and the fact that *p27*^*Kip1*^ and *Skp2* are regulated by other mechanisms in addition to Notch signaling. Next, to test the premise that *p27*^*Kip1*^ and *Skp2* expression are under Notch-mediated transcriptional repression and activation, respectively, we examined their proximal promoters for the presence of Hes1/CSL binding sites. Genes targeted by Notch signaling for repression, such as *p27*^*Kip1*^, contains C-box or class C site (CACGCG) elements where Hes1 forms transcriptional repression complexes (Fig 3B) [23]. Conversely, the direct target of Notch genes, such as *Hes1*/*Skp2*, contains CSL binding sites (TGGGAA) where NICD forms a complex for transcriptional activation (Fig 3E) [24]. To demonstrate that these sites were occupied by Hes1/CSL in MG, and therefore were targets of Notch-mediated transcriptional regulation, we carried out ChIP analyses of *p27*^*Kip1*^/*Skp2* promoters using Hes1/CSL antibodies (Fig 3D and 3F). We observed that Hes1 and CSL antibodies precipitated complexes from MG nuclei, containing DNA sequences corresponding to *p27*^*Kip1*^ (Fig 3D) and *Skp2* (Fig 3F) proximal promoters, the class C site and CSL binding sites, respectively. These DNA sequences with specific binding sites were absent in the ChIP assays carried out using IgG. Together, these observations suggested that both *p27*^*Kip1*^ and *Skp2* are targeted by Notch signaling during MG activation.

**Fig 4:**
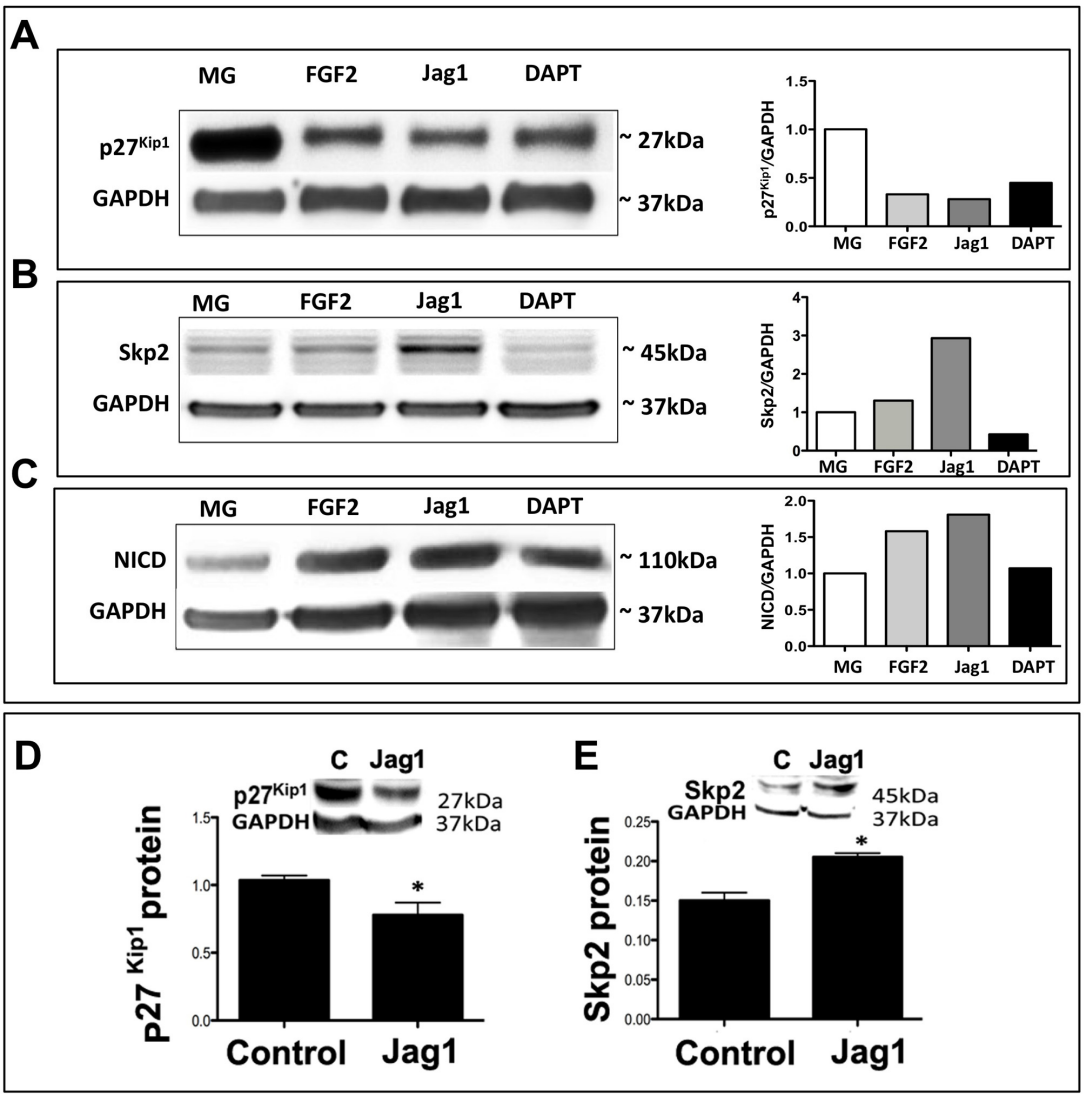
Post-translational influence of Notch signaling on p27^Kip1^ and Skp2 expression. **(A)** Western analyses revealed robust levels of p27^Kip1^ in enriched MG, which relatively decreased in FGF2/Jag-1 groups and increased in cells treated with DAPT. **(B)** In contrast, relative to that of p27^Kip1^, levels of Skp2 were less in MG but increased in cells treated with FGF2/Jag-1 and decreased in the DAPT group. **(C)** An increase and a decrease in protein levels of NICD were observed in the presence of FGF2/Jag1 and DAPT, respectively, indicating that Notch signaling was perturbed. Levels in graphs represent the arbitrary scanning units and all protein levels were normalized to GAPDH. **(D, E)** Quantitative Western analysis revealed a significant decrease and increase in levels of p27^Kip1^ (d) and Skp2 (e) protein levels in Jag1-treated groups, respectively, versus controls (FGF2), confirming the qualitative analyses carried out on the pooled samples (seen in “A”). *p<0.05. Data are mean ± SEM.

Next, we examined whether or not Notch signaling influenced p27^Kip1^ and Skp2 expression post-translationally. We examined levels of these proteins in response to perturbation of Notch signaling against the backdrop of their levels in enriched MG by Western analyses, carried out on pooled samples of cells/neurospheres because of the cell number limitations (Fig 4). A semi-quantitative analysis of the intensity of immunoreactive bands corresponding to specific protein levels in different conditions with respect to those at E18 revealed the following: robust levels of p27^Kip1^ were observed in enriched MG, which decreased by more than 3 fold in FGF2 and Jag-1-treated groups (p27^Kip1^/GAPDH: 1:00 versus 0.30 and 0.25, respectively) (Fig 4A). p27^Kip1^ levels remained decreased in DAPT-treated group relative to that in E18. However, when compared to Jag-1-treated groups, p27^Kip1^ levels were observed to be relatively higher (p27^Kip1^/GAPDH: 0.25 versus 0.35). In contrast, levels of Skp2 increased in FGF2 and Jag-1 groups, compared to that in E18 (Skp2/GAPDH: 1:00 versus 1.30 and 2.93, respectively) (Fig 4B). Skp2 levels in DAPT-treated groups remained lower versus all groups; they were more than 3 fold lower when compared to that in the FGF2 and Jag-1-treated groups (Skp2/GAPDH: 0.42 versus 1.30 and 2.93, respectively). That Notch signaling was perturbed in the experimental paradigm was demonstrated by an increase in NICD levels in FGF2/Jag-1-treated groups, compared to those in MG (Fig 4C). The decrease in NICD levels in response to DAPT treatment was observed in relation to FGF2/Jag-1 treatment. The detection of NICD in the presence of DAPT suggested an altered post-translational regulation for persistent Notch signaling, presumably required for the maintenance of glial phenotype. For example, the loss of function of Fbxw7 (F-box- and WD-repeat domain containing protein 7), which is required for targeted degradation of NICD, leads to accumulation of NICD with skewed differentiation of neural stem cells into astrocytes [40]. The accumulation of NICD may directly affect *Skp2* through transcriptional regulation and maintain its levels (Fig 3A) but not Skp2 protein, which might undergo normal degradation to have lower levels (Fig 4B, graph). To address whether or not the lack of difference in relative levels of p27^Kip1^ and Skp2 proteins in FGF2 and Jag-1 groups were bonafide or due to the pooling of cells, we carried out quantitative Western analysis. Because of cell limitations, particularly in the DAPT group, where size and numbers of neurospheres were low, the analysis included only two groups. We observed a significant decrease and increase in levels of immunoreactivities corresponding to p27^Kip1^ (Fig 4D) and Skp2 (Fig 4E) in the Jag1 group, respectively, compared to the FGF2 group. Together, these observations suggested that Notch signaling-mediated transcriptional and post-translational regulation of p27^Kip1^ and Skp2 might play a role in the activation of MG.

### Involvement of *p27*^*Kip1*^ in Notch-dependent activation of MG

**Fig 5:**
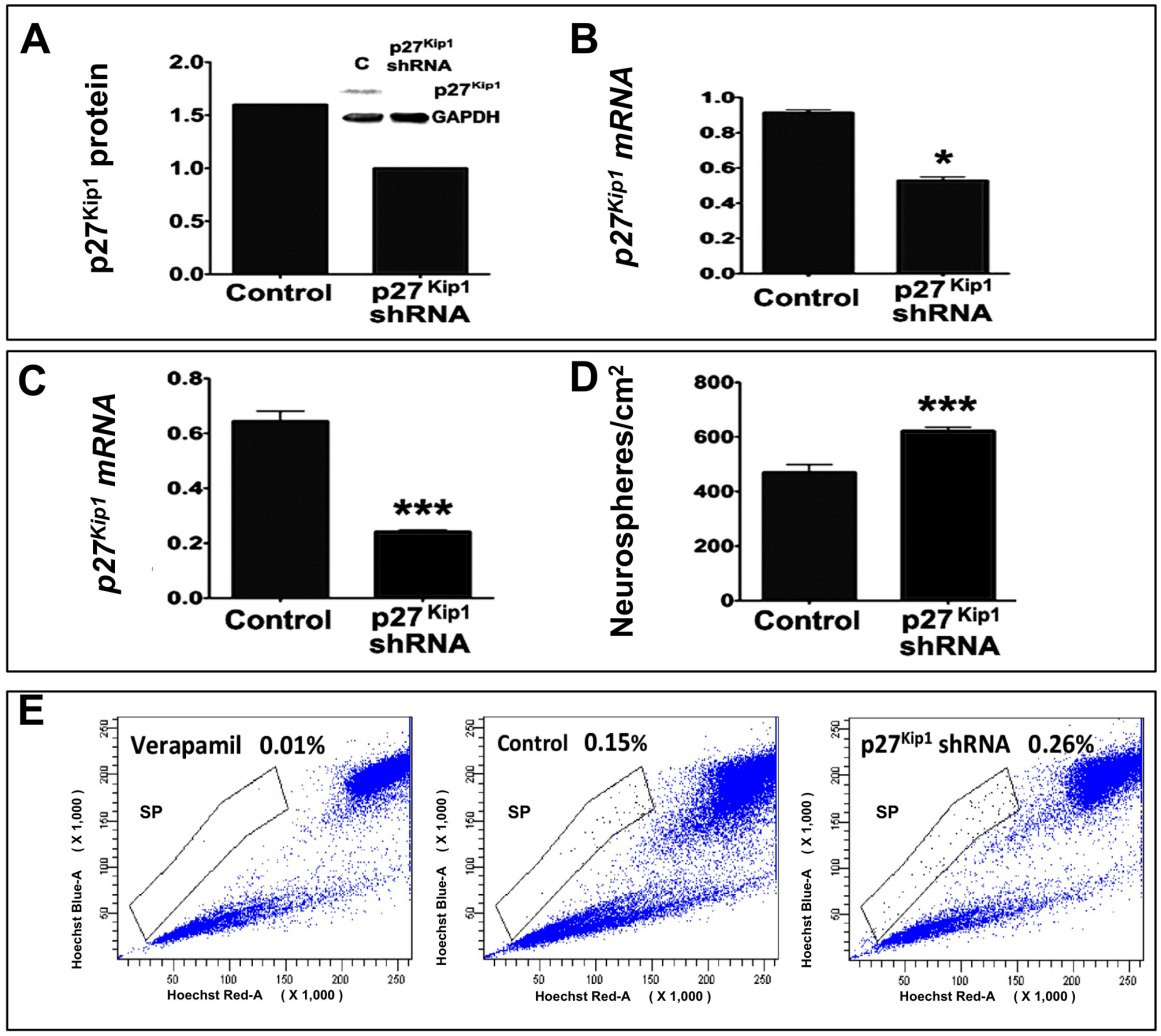
Effects of p27^Kip1^ loss of function on MG activation in neurosphere assays. **(A, B)** Validation experiments for the p27^Kip1^ loss of function approach in C6 glioma indicated a significant decrease in levels of p27^Kip1^ protein (a) and transcripts (b) in cells transduced with *p27*^*Kip1*^ shRNA lentivirus, compared to those transduced with control lentivirus. **(C, D)** MG transduced with *p27*^*Kip1*^ shRNA lentivirus and cultured in the presence of Jag1 displayed a significant decrease in *p27*^*kip1*^ transcript levels (c) and generated significantly more neurospheres (d) compared to controls (FGF2-treated controls). **(E)** Hoechst dye efflux assay revealed roughly a 2-fold increase in the number of SP cells in the *p27*^*Kip1*^ shRNA transduced MG-derived neurospheres compared to controls. The near absence of SP cells in the Hoechst dye efflux assay, carried out in the presence of verapamil, validated the assay. *p<0.05 and ***p<0.0005. All proteins and mRNA were normalized to GAPDH. Data are mean ± SEM. Experiments were carried out three times in triplicates with 10–12 PN10 rats for MG enrichment/batch.

To test whether or not *p27*^*Kip1*^ is involved in Notch-dependent activation of MG, we examined the effects of Jag1 on the activation of MG where levels of p27^Kip1^ were experimentally attenuated. The loss of function (LOF) approach was preferred over the gain of function (GOF) approach, as the latter may lead to non-specific effects. Given the cell number limitation that precluded Western analysis in MG, we tested the efficiency and specificity of p27^Kip1^ LOF in C6 glioma cells. We observed that *p27*^*Kip1*^ shRNA lentivirus-mediated transduction caused a reproducible and significant decrease in levels of both proteins and transcripts corresponding to p27^Kip1^, compared to controls (Fig 5A and 5B). Next, enriched MG were transduced with *p27*^*Kip1*^ shRNA or control lentivirus and subjected to neurosphere assays in the presence of Jag1 peptide. We measured the generation frequency of neurospheres and the proportion of SP cells to evaluate the effects of the p27^Kip1^ LOF on proliferation and stem cell property of the activated MG, respectively. We observed a significant decrease in *p27*^*Kip1*^ transcripts levels in the p27^Kip1^ LOF group, compared to controls (Fig 5C), which was correlated with a significant increase in the number of neurospheres (Fig 5D), suggesting that the loss of p27^Kip1^ expression further facilitated Notch-mediated generation of neurospheres. The neurospheres were subjected to HDEA to measure the proportion of SP cells by flow cytometry. The proportion of MG-SP cells was observed to have almost doubled in the p27^Kip1^ LOF group, compared to controls (0.26% versus 0.15%), suggesting an increase in the number of MG cells with stem cell property when the function of p27^Kip1^ was compromised (Fig 5E).

**Fig 6:**
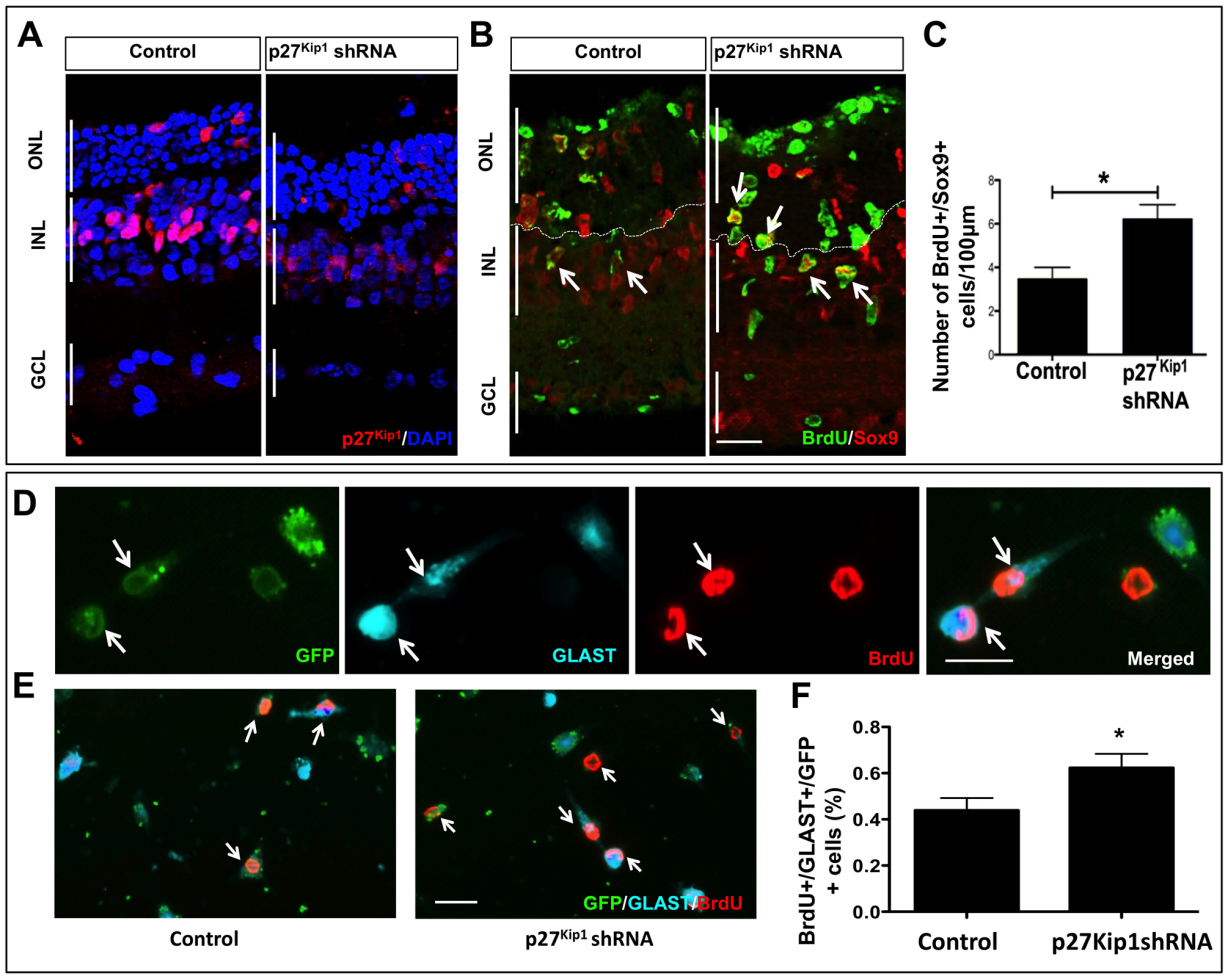
Effects of p27^kip1^ loss of function on MG activation in retinal explants. **(A)** Adult rat retinal explants were transduced overnight with *p27*^*Kip1*^ shRNA or control lentivirus and cultured for 5 days in retinal culture medium. Immunohistochemical analysis of retinal explants revealed a decrease in immunoreactivities corresponding to p27^Kip1^ in the p27^Kip1^ LOF group. **(B, C)** Immunocytochemistry and quantification of immunoreactive cells revealed an increase in the proportion of Sox9^+^BrdU^+^ cells in the p27^Kip1^ LOF group, compared to controls. **(D)** To determine the specificity of the p27^Kip1^ LOF approach, retinal explants were co-transduced with p27^Kip1^ shRNA and GFP lentiviruses/GFP lentivirus. Explants were dissociated, and transduced MG were identified as GFP^+^ cells which had incorporated BrdU and displayed immunoreactivities to GLAST. The lentivirus transduction efficiency is illustrated by transduced GFP^+^ cells (arrows), co-localized with immunoreactivities corresponding to BrdU and GLAST. **(E)** Immunocytochemical analysis and quantification of GFP^+^ BrdU^+^ GLAST^+^ cells revealed their proportion to be significantly higher in the p27^Kip1^ LOF group versus controls. p<0.005. Scale bar = 25μm. Data are mean ± SEM. Experiments were carried out three times in triplicates with 4–6 retina per group for *ex-vivo* perturbation. Broken lines distinguish the outer nuclear layer from inner nuclear layer.

Next, to determine that the Notch-dependent effects on the activation of enriched MG is not a function of the enrichment protocol that entails the exposure of MG to hypoxia and prolonged culture conditions, we carried out p27^Kip1^ LOF experiments in adult retinal explants, where *in vivo* cell-cell interactions are maintained and functional [14, 39]. Adult SD rat retinal explants were transduced with *p27*^*Kip1*^ shRNA or control lentivirus and cultured in retinal culture medium. BrdU was added in the last 8 hours before the termination of the culture [14]. Immunohistochemical analyses of retinal explants revealed a decrease in immunoreactivities corresponding to p27^Kip1^ in the p27^Kip1^ LOF group, compared to controls (Fig 6A). In contrast, we observed an increase in immunoreactivities corresponding to Sox9 and those detecting the incorporated BrdU, compared to controls (Fig 6B). These immunoreactivities, representing MG and proliferating cells, respectively, were largely confined to the inner nuclear layer and were co-localized. Since proliferating MG tend to migrate in retinal explant or in response to injury *in vivo*, some of these cells were also localized in the ONL [14]. Quantification of Sox9^+^ BrdU^+^ cells (= activated MG), revealed their numbers to be significantly increased in the p27^Kip1^ LOF group versus controls (Fig 6C). To further determine the specificity of the LOF approach, we co-transduced retinal explants with *p27*^*Kip1*^ shRNA lentivirus+GFP lentivirus/GFP lentivirus and cultured them as above. Explants were dissociated, transduced MG were identified as GFP epifluorescent cells, which co-expressed immunoreactivities corresponding to BrdU and glutamate/aspartate transporter (GLAST), an MG-specific marker (Fig 6D). Quantification of GFP^+^ GLAST^+^ BrdU^+^ cells revealed a significantly higher proportion in the p27^Kip1^ LOF group versus controls, suggesting along with the above results, that the loss of p27^Kip1^ function facilitates Notch signaling-mediated activation of MG (Fig 6E). Rare BrdU^+^ cells that were negative for MG markers, were excluded from quantification as they could represent microglia.

### Involvement of *Skp2* in Notch-dependent activation of MG

**Fig 7:**
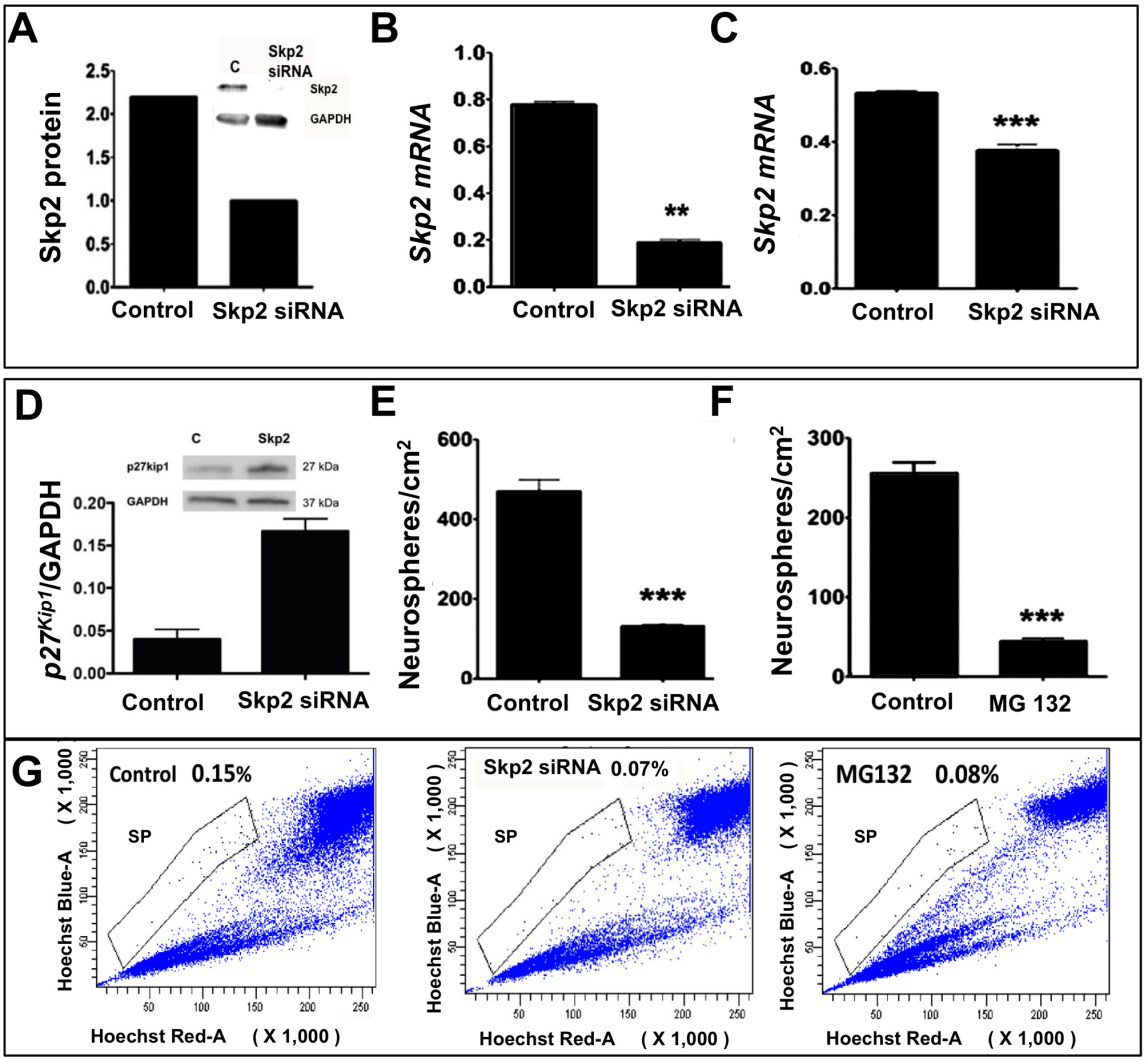
Effects of *Skp2* loss of function on MG activation in neurosphere assays. **(A, B)** Validation experiments for the Skp2 loss of function approach in C6 glioma revealed a significant decrease in Skp2 protein (a) and transcript (b) levels in cells transduced with *Skp2* siRNA lentivirus, compared to controls. **(C)** MG transduced with *Skp2* siRNA lentivirus and cultured in the presence of Jag1 displayed a significant decrease in *Skp2* transcript levels, compared to controls (FGF2-treated controls). **(D, E)** *Skp2* siRNA-transduced cells expressed higher levels of p27^kip1^ protein (D) and generated significantly fewer neurospheres (E), compared to controls. **(F)** Additionally, MG generated fewer neurospheres in the presence of proteosome inhibitor, MG132, versus controls. **(G)** Hoechst dye efflux assay revealed roughly half the number of SP cells in MG-derived neurospheres transduced with *Skp2* siRNA lentivirus, compared to controls. A similar decrease in SP cell number was observed in cells treated with MG132, compared to controls. **p<0.005 and ***p<0.0005. All protein and mRNA levels were normalized to GAPDH. Data are mean ± SEM. Experiments were carried out three times in triplicates with 10–12 PN10 rats for MG enrichment/batch.

Next, we used the LOF approach, as described above, to determine whether or not Skp2 was involved in Notch-dependent activation of MG. First, we examined the efficiency and specificity of the Skp2 LOF approach in C6 glioma cells, where *Skp2* siRNA lentivirus-mediated transduction led to a reproducible decrease in Skp2 protein (Fig 7A) and transcript (Fig 7B) levels, compared to controls. Second, when enriched MG were transduced with *Skp2* siRNA or control lentivirus and subjected to neurospheres assay in the presence of Jag1, a significant decrease in *Skp2* transcripts levels was observed, compared to controls (Fig 7C). Correspondingly, we observed an increase in the levels of p27^kip1^ protein in *Skp2* siRNA-treated neurospheres, compared to controls, demonstrating the regulatory influence of Skp2 on p27^kip1^ expression (Fig 7D). The decrease in *Skp2* expression was correlated with a significant decrease in numbers of neurospheres (Fig 7E), suggesting that the reduction of *Skp2* expression abrogated Notch-mediated generation of neurospheres. Since Skp2 mediates proteasomal degradation of target proteins such as p27^Kip1^ [24], it was expected that the pharmacological inhibition of proteasomes would have similar effects as the inhibition of Skp2 expression. To test this premise, enriched MG were subjected to neurosphere assay in the presence of proteasomal inhibitor MG132 [41]. The presence of MG132 led to a significant decrease in the generation of neurospheres, compared to controls, suggesting that the effects of Notch signaling at the post-transcriptional levels involved proteasomal degradation of proteins (Fig 7F). Third, we carried out HDEA on *Skp2*/control siRNA lentivirus-transduced MG-derived neurospheres. We observed 0.15% of MG-SP cells in Jag1-treated neurospheres, the proportion of which decreased to 0.07% and 0.08% when MG were pre-transduced with *Skp2* siRNA lentivirus or cultured in the presence of MG132, respectively (Fig 7G).

**Fig 8:**
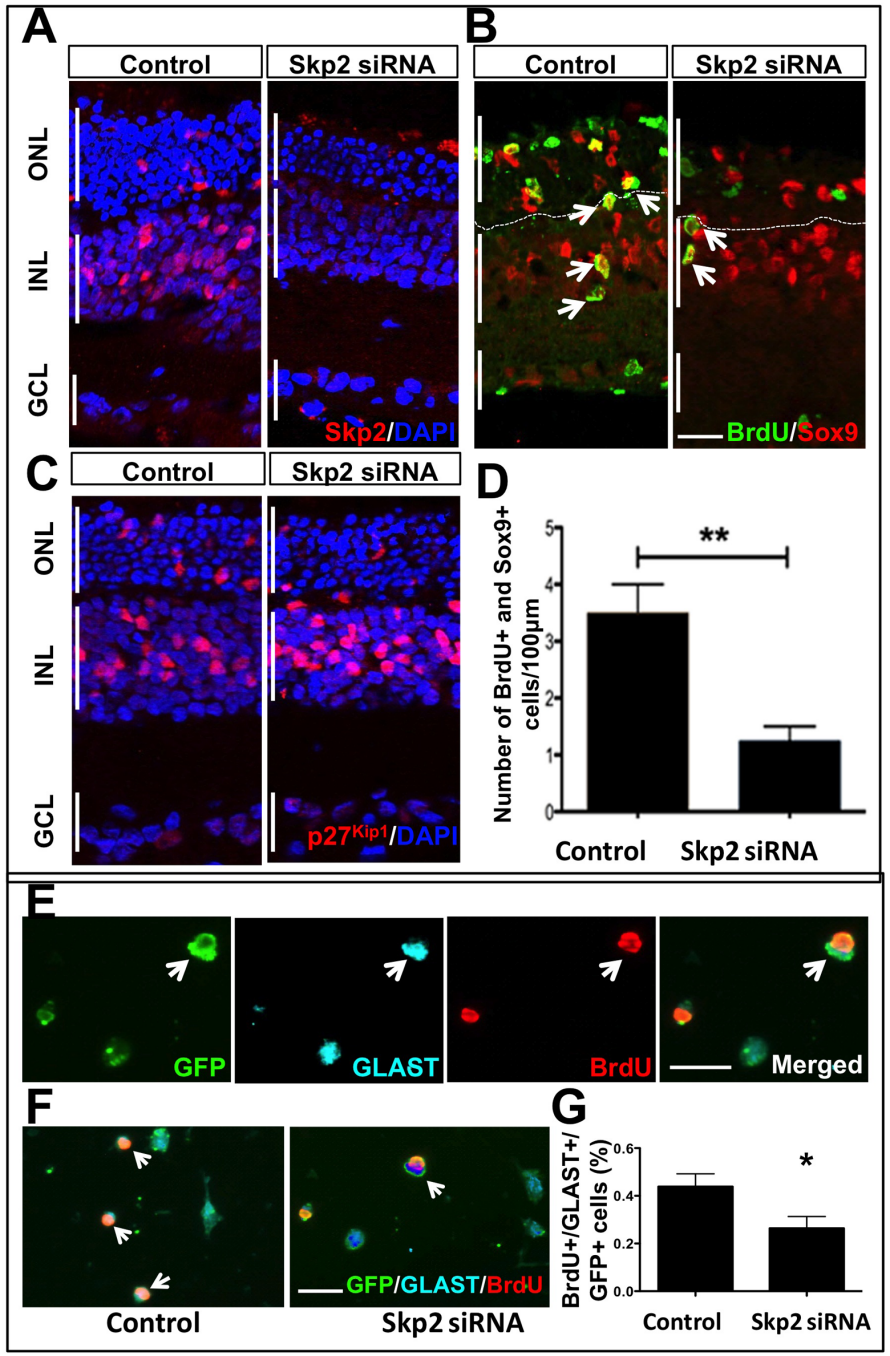
Effects of Skp2 loss of function on MG activation in retinal explants. **(A)** Immunohistochemical analyses of lentivirus transduced retinal explants revealed a decrease in immunoreactivities corresponding to Skp2 in the Skp2 LOF group versus controls. **(B, C)** Immunohistochemical analysis lentivirus transduced retinal explants (b) revealed significant decrease in the number of Sox9^+^ BrdU^+^ cells (c) in *Skp2* LOF groups, compared to controls. **(D)** Examination of these explants revealed increased in the number of cells expressing p27^kip1^ immunoreactivities in Skp2 LOF group versus controls. **(E)** To determine the specificity of the Skp2 LOF approach, retinal explants were co-transduced with Skp2 shRNA and GFP lentiviruses/GFP lentivirus. Explants were dissociated, and transduced MG were identified as GFP^+^ cells which had incorporated BrdU and displayed immunoreactivities to GLAST. The lentivirus transduction efficiency is illustrated by transduced GFP^+^ cells (arrows), co-localized with immunoreactivities corresponding to BrdU and GLAST. **(E)** Immunocytochemical analysis and quantification of GFP^+^ BrdU^+^ GLAST^+^ cells revealed their proportion to be significantly higher in the Skp2 LOF group versus controls. p<0.005. Scale bar = 25μm. Data are mean ± SEM. Experiments were carried out three times in triplicates with 4–6 retina per group for *ex-vivo* perturbation. Broken lines distinguish the outer nuclear layer from inner nuclear layer.

Lastly, to rule out that the observed effects were peculiar to enriched MG, Skp2 LOF experiments were carried out on retinal explants, as described above, where the transduction with *Skp2* siRNA led to a decrease in Skp2 immunoreactivities in retinal explants compared to controls (Fig 8A). In contrast to the results obtained by p27^Kip1^ LOF approach (Fig 6A), immunohistochemical analysis of transduced explants revealed a significant decrease in the number of Sox9^+^BrdU^+^ cells in Skp2 LOF groups, compared to controls, revealing the negative influence of Skp2 LOF on the Notch-mediated activation of MG (Fig 8B and 8C). That the stabilized p27^Kip1^ might have mediated the inhibitory effects was revealed by the increase in p27^Kip1^ immunoreactivities in Skp2 siRNA lentivirus transduced explants versus controls (Fig 8D). Skp2 LOF experiments involving co-transduction with GFP lentivirus to determine the specificity of the approach as above (Fig 8E) revealed a significant decrease in the number of GFP^+^ GLAST^+^ BrdU^+^ cells, versus controls (Fig 8F). Together, these observations suggested that the Notch signaling-mediated accentuation of Skp2 expression was involved in MG activation.

## Discussion

A variety of approaches involving different species suggests that MG, besides maintaining retinal homeostasis, may act as progenitors, capable of supporting neurogenesis in adults [1–3]. The evidence emerging from teleosts, particularly transgenic zebrafish, is robust for the neurogenic potential of MG; they serve as progenitors for photoreceptors during normal development and are recruited in response to injury for regeneration [2, 3]. While such regenerative properties of MG were not observed in the mammalian retina, they have been observed to respond to injury by re-entering the cell cycle [42,43]. Given their regenerative capacity in lower vertebrates and biochemical resemblance with radial glia, examination of MG enriched from rodent retina revealed that a subset of these cells possessed the ability to self renew and differentiate along the neuronal lineage [5]. It was observed that the display of stem cell properties by MG *in vitro* was accompanied by the activation of Notch signaling, and that its inhibition significantly compromised the abilities of these cells to generate clonal neurospheres in culture [5] or incorporate BrdU *in vivo* in response to injury [5,14]. The involvement of Notch signaling in the regulation of MG is also observed in zebrafish regeneration, where the temporal sequence of the activation of Notch signaling is better understood than in mammals [2]. For example, molecular examination of *gfap-EGFP*-labeled MG, enriched after different time intervals following a light induced lesion in transgenic zebrafish, revealed that during the initial stages of the injury, when MG have not yet divided, the indices of Notch signaling were decreased [44]. However, following injury, a stage associated with MG division, the expression of the components of Notch signaling were up-regulated, suggesting an association of Notch signaling with MG proliferation, as observed in mammals.

**Fig 9:**
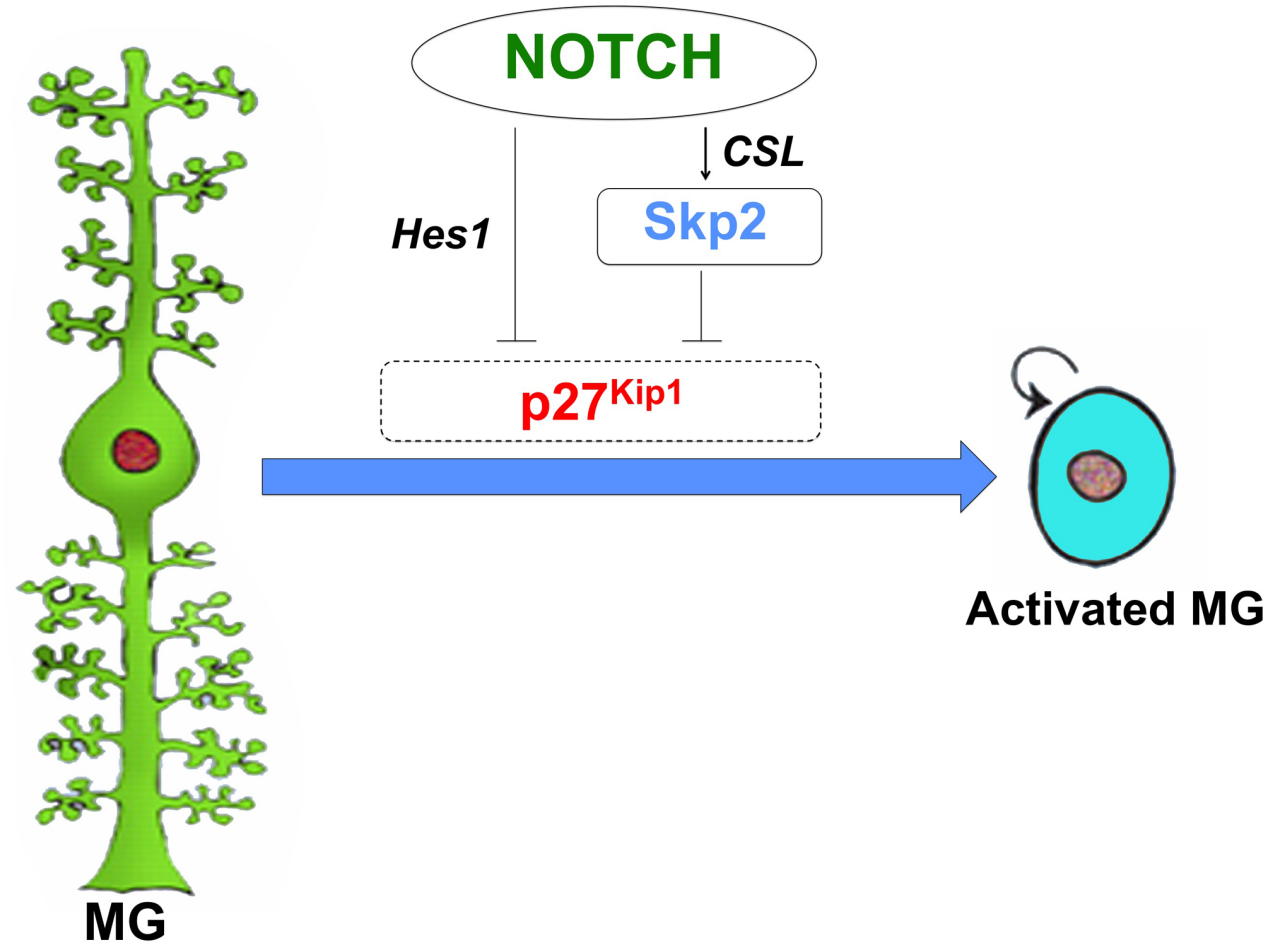
Notch signaling regulates p27^Kip1^ expression to influence the activation of MG. p27^Kip1^ expression persists in MG as one of their characteristic molecular features. The activation of Notch signaling in quiescent MG leads to a decrease in the expression of p27^Kip1^ through Hes1-mediated transcriptional repression and Skp2-mediated post-translational inhibition, promoting the G1-S transition.

Among the multiple signaling pathways that are recruited for the development and differentiation of the retina, the one that is more appropriately placed, enabling MG to sense and respond to injury, is the Notch signaling. It is used in its classical function to regulate cell commitment and also to promote proliferation of progenitor/precursor populations, as observed in a variety of tissues [45]. In the developing retina, Notch receptor activation ensures that a subset of progenitors remain uncommitted during early histogenesis, presumably to sustain the prolonged cell type-specific differentiation. During late histogenesis, Notch receptors are recruited to promote differentiation of MG, and therefore, when Notch signaling is experimentally perturbed, MG generation is adversely impacted [46]. How does Notch function change from maintaining progenitor populations during early histogenesis to promoting differentiation of glia in late histogenesis remains poorly understood. It is thought that the strengthening of the JAK-STAT pathway in retinal progenitors, at the behest of cytokines secreted by differentiating cells during late histogenesis, may underlie the gliogenic effects of Notch signaling [47,48]. Evidence based on the spatial localization of the expression of Notch receptor in adult retina suggests that Notch signaling may remain active in MG or at least in a subset of these cells [49–52]. The role of Notch signaling in adult MG remains unexplored, however, current observations suggest that it is involved in cell cycle regulation to sustain the regenerative role of MG [1, 2]. For example, experimental accentuation of Notch signaling in S334 rats in the beginning of rod photoreceptor degeneration results in MG proliferation and migration to the ONL where a minor subset of these cells begin to express markers corresponding to degenerated neurons [14]. Similar influence of Notch signaling on MG proliferation and regenerative potential has been observed in chicks [51–53]. Here, we have demonstrated the possible mechanism by which the activation of Notch signaling is linked to MG proliferation. While *myc* and *cyclins* have been demonstrated to be the direct targets of Notch signaling for G1-S transition in Notch-activated cells [54], it is likely that the proliferative influence in MG is mediated through p27^Kip1^ (Fig 9). p27^Kip1^ is up regulated during histogenesis to promote committed progenitors/precursors exit from the cell cycle [28, 36]. Its expression attenuates in differentiated cells but persists in MG as one of its characteristic molecular features [5, 55]. While the maintenance of high levels of p27^Kip1^ expression in terminally differentiated cells is rather enigmatic, it could be suggested that in MG, it is employed to keep their evolutionarily conserved propensity to re-enter the cell cycle [1]. In this model, Notch signaling, influenced by adjacent cells and/or by Mash-1 based cell intrinsic machinery observed in zebrafish [56], abrogates the levels of p27^Kip1^ proteins by acting through the *Notch-p27*^*Kip1*^ and *Notch-Skp2-p27*^*Kip1*^ axes. The recruitment of these axes, one inhibiting the transcription of *p27*^*Kip1*^ and the other leading to the Skp2-mediated degradation of p27^Kip1^ proteins, may have evolved for a rapid and sustained proliferative response of MG to injury.

Our observations shed light on the possible mechanism of Notch signaling-mediated activation of MG, and not when it is employed or how it is linked to the neural conversion of MG. If one leans upon the evidence emerging from zebrafish [2, 20, 21, 56], it might not be implausible to consider that sometime during the early response of MG to injury, Notch signaling is recruited to facilitate a cell proliferation response. This window is likely to be narrow and with the change in context, spatial (migration out of the INL) and/or temporal, the effects of Notch signaling would also change. If Notch signaling persists when MG are poised for neuronal conversion, it might be deleterious for the regenerative process, given the inhibitory influence of Notch signaling on the expression of pro-neural genes. Therefore, inhibiting Notch signaling at this stage has been observed to expand the regenerative field [56] as well as neuronal conversion of MG [1,2]. Similar temporal manipulation of Notch signaling, directly or indirectly, by targeting the Notch-dependent molecular axes, may help recruit MG for regeneration in the mammalian retina.
